# Supplementary material for: Association between oral microbiome and seven types of cancers in East Asian population: a two-sample Mendelian randomization analysis
Source: Front Mol Biosci. 2023 Nov 21;10:1327893. doi: 10.3389/fmolb.2023.1327893 (PMC10702768; doi:10.3389/fmolb.2023.1327893)
Supplement: Supplementary file 1 [file DataSheet1.ZIP › Supplementary Materials/MR plots for tongue/tongue═╝/Gastric cancer/pheno.2338_to_gastric cancer_forest.pdf]

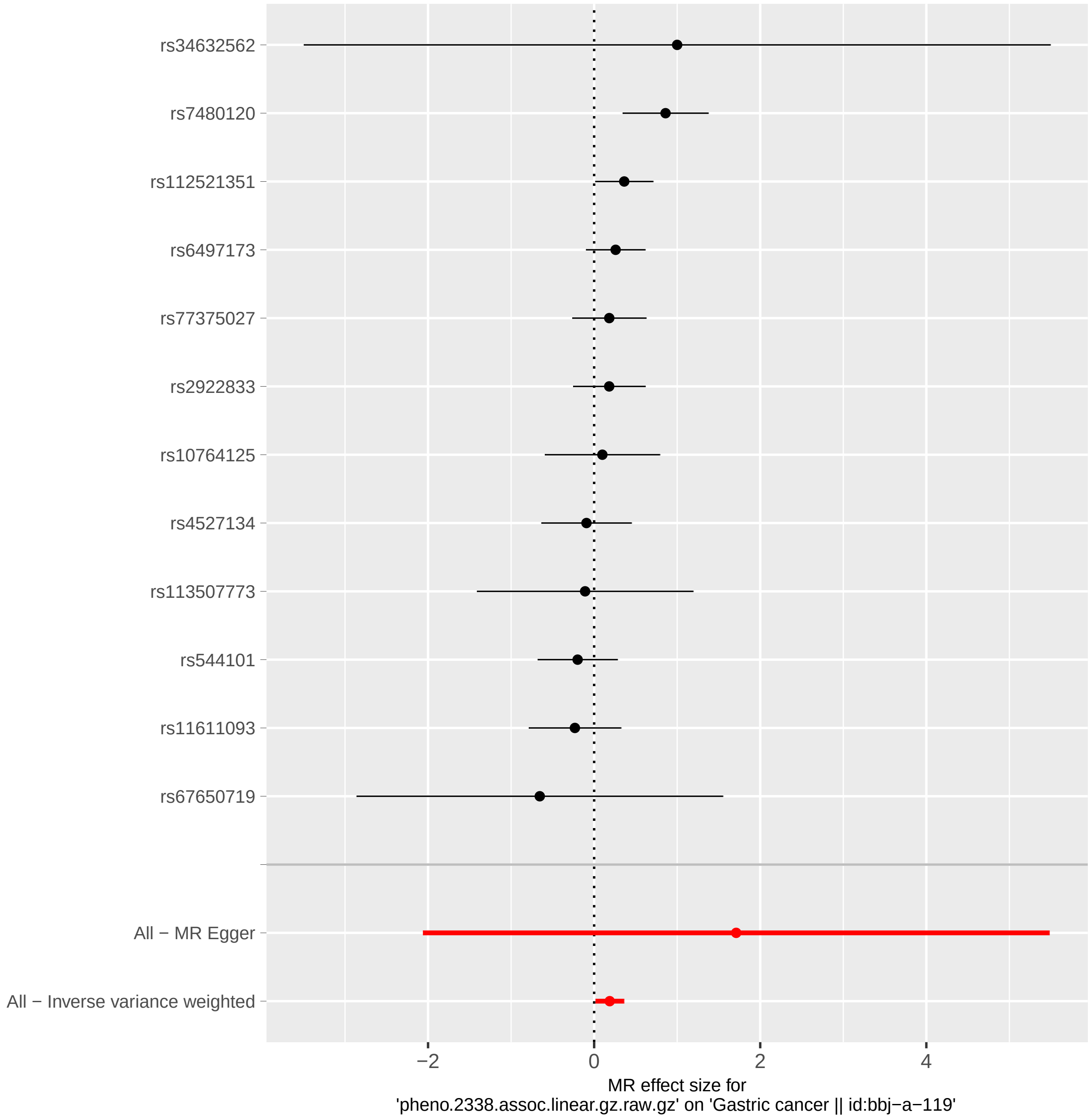
rs34632562
rs7480120
rs112521351
rs6497173
rs77375027
rs2922833
rs10764125
rs4527134
rs113507773
rs544101
rs11611093
rs67650719
All − MR Egger
All − Inverse variance weighted
−2
0
2
4
MR effect size for
'pheno.2338.assoc.linear.gz.raw.gz' on 'Gastric cancer || id:bbj−a−119'
